# Nationwide Trends in Hospitalizations and Clinical Outcomes for Meningitis and Encephalitis: A 10-year Swiss Population-based Study

**DOI:** 10.1093/ofid/ofag350

**Published:** 2026-06-05

**Authors:** Lennart Essmann, Claudia Gregoriano, Philipp Schuetz, Anna Conen, Alexander Kutz

**Affiliations:** Department of Internal Medicine and Primary Care Medicine, Cantonal Hospital Aarau, Medical University Clinic, Aarau, Switzerland; Department of Cardiology, Cantonal Hospital Aarau, Aarau, Switzerland; Department of Internal Medicine and Primary Care Medicine, Cantonal Hospital Aarau, Medical University Clinic, Aarau, Switzerland; Department of Internal Medicine and Primary Care Medicine, Cantonal Hospital Aarau, Medical University Clinic, Aarau, Switzerland; Medical Faculty, University of Basel, Basel, Switzerland; Medical University Department, Clinic for Endocrinology, Diabetes and Metabolism, Cantonal Hospital Aarau, Aarau, Switzerland; Department of Internal Medicine and Primary Care Medicine, Cantonal Hospital Aarau, Medical University Clinic, Aarau, Switzerland; Medical University Department, Clinic for Infectious Diseases and Infection Prevention, Cantonal Hospital Aarau, Aarau, Switzerland; Department of Internal Medicine and Primary Care Medicine, Cantonal Hospital Aarau, Medical University Clinic, Aarau, Switzerland; Medical Faculty, University of Basel, Basel, Switzerland

**Keywords:** central nervous system infections, encephalitis, in-hospital mortality, meningitis, nationwide cohort, Switzerland

## Abstract

**Background:**

Meningitis and encephalitis are severe infections of the central nervous system (CNS) causing substantial morbidity and mortality. Contemporary nationwide data on epidemiological trends and outcomes are limited.

**Methods:**

We conducted a nationwide, retrospective cohort study using hospital discharge data from the Swiss National Health Registry. All hospitalizations for infectious meningitis and encephalitis between January 2012 and December 2021 were included. We assessed temporal and age-specific trends, pathogen distributions, and in-hospital outcomes using descriptive statistics and multivariable regression analysis.

**Results:**

Among 23 426 identified hospitalizations (10 160 for meningitis; 13 266 for encephalitis), the overall incidence of CNS infections increased from 22 to 34 per 100 000 inhabitants between 2012 and 2019, followed by a marked decline in 2020–2021. For meningitis, this decline was driven by reductions in pneumococcal (∼65%) and enteroviral (∼90%) infections. Enteroviral and streptococcal meningitis rates were highest in children younger than 10 years, whereas *Streptococcus pneumoniae, Listeria monocytogenes*, tick-borne encephalitis virus, and varicella-zoster virus infections were more frequent in adults older than 50 years. Compared with viral meningitis, bacterial meningitis was associated with higher odds of intensive care unit admissions (odds ratio [OR], 8.40; 95% confidence interval [CI], 7.29 to 9.67) and in-hospital mortality (OR 4.23; 95% CI, 2.95 to 6.05). Similar risk patterns were observed for bacterial encephalitis.

**Conclusions:**

Nationwide hospitalization rates for CNS infections showed distinct temporal and age-specific patterns. Bacterial infections, particularly among older adults, were associated with substantially worse outcomes, underscoring the importance of early diagnosis, optimized treatment, and preventive strategies, including vaccination.

## Teaser

This nationwide study (2012–2021) identified distinct temporal and age-specific trends in hospitalizations for central nervous system infections in Switzerland. Bacterial etiologies, particularly among older adults, were linked to worse clinical outcomes, emphasizing the importance of early diagnosis and treatment, including optimization of prevention measures.

Meningitis and encephalitis represent severe, life-threatening infections of the central nervous system (CNS), which are most frequently precipitated by bacterial and viral pathogens. Clinical manifestations range from self-limited febrile illness to fulminant neurologic injury and death [1–3]. Owing to their rapid progression and potential for long-term disability and fatality, these conditions remain a substantial public health concern. In 2019, an estimated 2.51 million cases of meningitis and 236 000 related deaths were reported globally [3]. Although mortality from bacterial meningitis in Europe has declined by more than 50% since 1990, it remains a leading cause of CNS-related death, particularly among young children and older adults [3]. In response, the World Health Organization launched the “Defeating Meningitis by 2030” global roadmap in 2021, aiming to eliminate bacterial meningitis epidemics, reduce vaccine-preventable cases, and mortality [4].

In Switzerland, demographic aging, increasing comorbidity burden, and evolving pathogen profiles pose new challenges for the prevention and management of CNS infections. Therefore, up-to-date epidemiological data are needed to inform vaccination strategies, clinical management, and resource planning. A recent Swiss study of 258 cases of meningitis and encephalitis between 2016 and 2018 provided important insights but was limited by sample size and restricted regional and temporal generalizability [5]. Although prior studies have investigated selected CNS infections or specific populations [1, 6–8], comprehensive population-based analyses across the full age spectrum that simultaneously assess pathogen distribution, temporal trends, and clinical outcomes remain scarce. Age is a key determinant of both susceptibility and prognosis. Children are particularly vulnerable to viral pathogens, especially enteroviruses, the leading cause of viral meningitis [1, 6, 9]. In contrast, older adults face a higher risk of bacterial infections caused by *Streptococcus pneumoniae* and *Listeria monocytogenes*, likely reflecting immunosenescence and a higher prevalence of predisposing comorbidities including immunosuppression [2].

In this nationwide study, we sought to characterize the epidemiology and clinical outcomes of CNS infections across all age groups in Switzerland. Specifically, we assessed pathogen-specific incidence rates (IRs), described age-related patterns, and compared in-hospital outcomes between bacterial and viral meningitis and encephalitis.

## METHODS

### Study Design and Data Source

We conducted a retrospective, nationwide cohort study including all hospitalizations for bacterial and viral meningitis and encephalitis in Switzerland between January 1, 2012 and December 31, 2021. Data were obtained from the Swiss Federal Statistical Office (Bundesamt für Statistik, Neuchâtel, Switzerland), which compiles pseudonymized discharge records from all acute care hospitals and provides near complete coverage of hospitalizations. The dataset contains individual-level information on patient demographics, diagnoses, procedures, and in-hospital outcomes. Diagnoses were coded according to the International Classification of Diseases, 10th Revision, German Modification (ICD-10-GM) (http://www.who.int/classifications/icd/en/), which was used to identify cases of meningitis and encephalitis.

The data were pseudonymized before analysis through a multistep deidentification process. The ethics committee of Northwestern and Central Switzerland (Ethikkommission Nordwest- und Zentralschweiz, EKNZ) declared that this study did not fall under the Human Research Act, as data were pseudonymized prior to analysis (EKNZ Project-ID: Req-2021-01379). Formal ethical approval was therefore not required. This study adhered to the “strengthening the reporting of observational studies in epidemiology” reporting guidelines [10].

### Case Ascertainment and Study Variables

We included patients of all ages hospitalized with bacterial or viral meningitis or encephalitis, identified by primary or secondary ICD-10-GM diagnosis codes. Pathogen-specific codes were used to classify etiologies (eg, G00.1 for pneumococcal meningitis, A87 for enteroviral meningitis, A32.1 in combination with G05.0 for *Listeria* encephalitis, and B00.4 for herpes simplex encephalitis). For pathogen-specific CNS analyses, only codes explicitly indicating CNS involvement were considered pathogen-attributable. While the analysis focused on bacterial and viral etiologies, fungal and parasitic pathogens were not separately classified in the ICD-10 search strategy; cases with these etiologies may nevertheless have been captured within “unspecified’ categories when only nonspecific diagnostic codes were assigned. Cases coded as meningoencephalitis were categorized as encephalitis to reflect clinical practice, where etiologic differentiation is often limited by overlapping clinical presentations and incomplete pathogen identification. Hospitalizations, including readmissions and interhospital transfers, were analyzed at the hospitalization level, with each admission treated as a separate episode. However, in accordance with SwissDRG rules, rehospitalization to the same hospital for the same medical reason within 18 days after discharge were consolidated into a single case. A complete list of ICD-10-GM codes is provided in Supplementary Table 1. Comorbidity burden was quantified using the Elixhauser comorbidity index [11], and frailty was assessed with the validated hospital frailty risk score [12].

### Outcomes

The primary in-hospital outcomes included in-hospital mortality, intensive care unit (ICU) admission, mechanical ventilation, length of stay (LOS), and discharge disposition. Outcomes were analyzed overall and stratified by age, sex, and comorbidity burden.

### Statistical Analysis

Baseline characteristics are presented as counts (%) or means (standard deviation [SD]). Annual IRs per 100 000 population were calculated overall and by pathogen group and 5-year age strata, using corresponding yearly population estimates from Switzerland as denominators. Temporal and age-specific trends in incidence and in-hospital case fatality rates (CFR), defined as the percentage of in-hospital deaths among hospitalized cases (in-hospital mortality), were visualized using locally weighted scatterplot smoothing (LOWESS). LOWESS smoothing was applied consistently across all trend figures to preserve continuous temporal and age-specific patterns without imposing categorical cutoffs that might obscure transitional trends. Trends over time and across age groups were assessed with the nonparametric Jonckheere-Terpstra test.

Given the marked decline in incidence after 2019, which may introduce nonmonotonic patterns, temporal analyses were conducted separately for the prepandemic period (2012–2019) and the entire study period (2012–2021) to ensure robustness of trend estimates. Less frequent pathogens were grouped as “other” to enhance interpretability. Age groups older than 90 years were excluded from smoothed trend curves owing to small sample sizes. Hospitalizations with a LOS >100 days were excluded to limit the influence of extreme outliers.

To examine associations between infection type and clinical outcomes, we used logistic regression for binary outcomes and linear regression for continuous outcomes. All models were adjusted for age, sex, and comorbidity burden. Results are reported as adjusted odds ratios (ORs) or mean differences with 95% confidence intervals (CIs). No correction for multiple testing was applied, and all statistical tests were two-sided with statistical significance set at *P* < .05. Analyses were performed using Stata, version 17.0. (StataCorp LLC, College Station, TX, USA).

## RESULTS

### Patient Characteristics

Between January 1, 2012, and December 31, 2021, we identified 23 426 hospitalizations for CNS infections in Switzerland, including 10 160 cases of meningitis (43.4%) and 13 266 cases of encephalitis (56.6%) (Supplementary Figure 1). The mean age was 36.2 (SD 27.3) years for meningitis and 53.9 (SD 22.8) years for encephalitis. Patients aged 65 years or older accounted for 18% of meningitis and 36% of encephalitis cases. Sex distribution was similar in both groups (54.6% males). Compared to patients with meningitis, those with encephalitis had a higher comorbidity burden and in-hospital frailty score. Additional characteristics are summarized in Table 1; diagnostic groupings by type of infection are provided in Supplementary Table 2.

**Table 1.** Baseline Characteristics of Patients With Meningitis and Encephalitis

	Total	Meningitis	Encephalitis	*P* Value
	N = 23 426	N = 10 160	N = 13 266	
Age, years, mean (SD)	46.2 (26.3)	36.2 (27.2)	53.9 (22.8)	<.001
Age categories, n (%)				
0–10 y	3468 (14.8)	2645 (26.0)	823 (6.2)	<.001
11–20 y	1434 (6.1)	813 (8.0)	621 (4.7)	
21–49 y	6569 (28.0)	3132 (30.8)	3437 (25.9)	
50–65 y	4730 (20.2)	1554 (15.3)	3176 (23.9)	
>65 y	7225 (30.8)	2016 (19.8)	5209 (39.3)	
Male, n (%)	12 790 (54.6)	5504 (54.2)	7286 (54.9)	.25
Elixhauser comorbidity index, mean (SD)	1.5 (1.9)	1.1 (1.7)	1.9 (1.9)	<.001
In-hospital frailty score categories, n (%)				
<5 points (low)	16 671 (71.2)	8228 (81.0)	8443 (63.6)	<.001
5–15 points (intermediate)	5848 (25.0)	1667 (16.4)	4181 (31.5)	
>15 points (high)	907 (3.9)	265 (2.6)	642 (4.8)	
Comorbidities, n (%)				
Obesity	285 (1.2)	106 (1.0)	179 (1.3)	.034
Diabetes mellitus	2048 (8.7)	607 (6.0)	1441 (10.9)	<.001
Atherosclerotic cardiovascular disease^a^	3150 (13.4)	1014 (10.0)	2136 (16.1)	<.001
Chronic pulmonary disease^b^	1160 (5.0)	312 (3.1)	848 (6.4)	<.001
URTI or ENT infections^c^	1204 (5.1)	741 (7.3)	463 (3.5)	<.001
LRTI^d^	1242 (5.3)	516 (5.1)	726 (5.5)	.18
Malignancy^e^	2392 (10.2)	1080 (10.6)	1312 (9.9)	.064
Immunodeficiency^f^	787 (3.4)	272 (2.7)	515 (3.9)	<.001
HIV infection	248 (1.1)	146 (1.4)	102 (0.8)	<.001
Splenectomy	30 (0.1)	26 (0.3)	4 (0.0)	<.001
Alcohol dependence	657 (2.8)	223 (2.2)	434 (3.3)	<.001
Liver disease, including cirrhosis	106 (0.5)	35 (0.3)	71 (0.5)	.031
Chronic kidney disease	1661 (7.1)	463 (4.6)	1198 (9.0)	<.001
Craniofacial anomalies	9 (0.0)	5 (0.0)	4 (0.0)	.46
Cerebrospinal fluid fistula	178 (0.8)	130 (1.3)	48 (0.4)	<.001
Presence of cochlear implant	12 (0.1)	7 (0.1)	5 (0.0)	.3

Abbreviations: ENT, ear, nose, and throat; HIV, human immunodeficiency virus; LRTI, lower respiratory tract infection; SD, standard deviation; URTI, upper respiratory tract infection.

^a^ASCVD (atherosclerotic cardiovascular disease) includes coronary artery disease, peripheral arterial disease, and cerebrovascular disease.

^b^Chronic pulmonary disease includes chronic obstructive pulmonary disease (COPD), asthma, and obstructive sleep apnea syndrome (OSAS).

^c^URTI and ENT infections include external and middle ear infections (eg, otitis media and mastoiditis) and acute upper respiratory infections (eg, sinusitis and pharyngitis).

^d^LRTI includes influenza, pneumonia, and bronchitis (ICD-10 J09–J22).

^e^Malignancy includes both hematologic cancers and solid tumors.

^f^Immunodeficiency was defined by ICD-10 codes: primary immunodeficiencies (D80–D84), agranulocytosis (D70), immunodeficiency after chemotherapy or immunosuppressants (D90), other immune disorders (D89), transplant status (Z94), and other specified immunodeficiencies (D84.8).

### Temporal Trends

The overall incidence of CNS infection-related hospitalizations increased by nearly 50% from 2012 to 2019 (from 22 to 34 per 100 000 inhabitants; *P*_trend_ < .01), followed by a marked decline in 2020 and 2021 (Table 2; Supplementary Figure 2).

**Table 2.** Temporal Trends in Incidence Rates of Meningitis and Encephalitis

Diagnosis	Years											*P* For Trend	
IR per 100 000 Inhabitants	2012	2013	2014	2015	2016	2017	2018	2019	2020	2021	Mean (SD)	≤ 2019	Overall
Overall	21.99	26.13	24.63	25.71	28.62	31.18	32.82	33.80	29.44	26.07	28.0 (3.8)	**<**.**01**	.**04**
Meningitis	11.18	13.48	12.03	12.47	12.55	14.09	14.01	14.90	8.69	8.61	12.2 (2.2)	.**01**	.79
Bacterial meningitis	2.83	3.47	3.70	3.71	3.56	4.07	3.32	4.01	2.88	2.52	3.4 (0.5)	.14	.79
*Haemophilus influenzae*	0.09	0.12	0.12	0.08	0.14	0.15	0.18	0.16	0.06	0.09	0.1 (0.0)	.**03**	.53
*Streptococcus pneumoniae*	0.72	0.87	0.77	0.81	0.71	0.95	0.76	0.77	0.27	0.30	0.7 (0.2)	.80	.24
*Streptococcus* spp.	0.28	0.53	0.64	0.56	0.46	0.63	0.41	0.69	0.48	0.36	0.5 (0.1)	.32	.93
*Staphylococcus* spp.	0.25	0.27	0.54	0.51	0.28	0.40	0.27	0.53	0.44	0.34	0.4 (0.1)	.46	.53
*Neisseria meningitidis*	0.29	0.37	0.27	0.35	0.30	0.38	0.43	0.25	0.19	0.06	0.3 (0.1)	.62	.24
Others	0.16	0.19	0.17	0.16	0.19	0.21	0.09	0.18	0.21	0.14	0.2 (0.0)	.80	.93
Viral meningitis	6.46	8.18	6.19	6.87	7.03	8.35	8.49	8.67	3.74	4.03	6.8 (1.8)	.**01**	.65
Enterovirus	1.50	2.37	1.10	2.41	2.24	3.49	3.67	3.86	0.30	0.77	2.2 (1.2)	.**01**	.65
Adenovirus	-	-	-	-	-	0.01	0.01	-	-	-	0.0 (0.0)	.32	.32
Herpes simplex virus	0.31	0.32	0.42	0.44	0.44	0.37	0.50	0.52	0.50	0.38	0.4 (0.1)	**<**.**01**	.**03**
Varicella-zoster virus	0.53	0.42	0.64	0.53	0.60	0.73	0.66	0.69	0.57	0.75	0.6 (0.1)	.**03**	.**03**
Others	0.01	0.01	-	0.02	0.01	0.02	-	-	-	-	0.0 (0.0)	1.00	1.00
Encephalitis	10.80	12.66	12.60	13.24	16.07	17.10	18.81	18.89	20.75	17.46	15.8 (3.3)	**<**.**01**	**<**.**01**
Bacterial encephalitis	0.87	0.98	1.18	1.36	1.90	1.57	1.60	1.98	1.53	1.64	1.5 (0.4)	**<**.**01**	**<**.**01**
*Listeria monocytogenes*	0.03	0.07	0.11	0.05	0.08	0.01	0.02	0.02	0.06	0.03	0.0 (0.0)	.22	.42
*Neisseria meningitidis*	-	0.04	0.04	-	-	0.02	0.04	-	0.02	0.02	0.0 (0.0)	.17	.**01**
*Mycobacterium tuberculosis*	0.04	0.01	0.04	-	0.01	0.04	0.02	-	0.02	0.07	0.0 (0.0)	.35	1.00
*Treponema pallidum*	-	0.01	0.01	-	-	-	-	-	0.02	-	0.0 (0.0)	.32	.60
Viral encephalitis	4.35	6.52	5.92	5.75	7.19	8.51	9.86	9.29	10.81	7.77	7.6 (2.0)	.**01**	**<**.**01**
Herpes simplex virus	0.93	0.91	1.43	1.13	1.09	1.57	1.22	1.37	1.32	0.95	1.2 (0.2)	.14	.42
Varicella-zoster virus	0.57	0.98	1.14	0.90	1.27	1.20	1.21	1.43	1.11	1.39	1.1 (0.3)	.**01**	.**03**
Tick-borne	1.34	2.79	1.53	1.72	2.72	3.77	5.26	3.86	6.28	3.72	3.3 (1.6)	.**01**	**<**.**01**
Arthropod-borne	0.15	0.25	0.22	0.34	0.54	0.37	0.56	0.60	0.64	0.44	0.4 (0.2)	**<**.**01**	**<**.**01**
Others	0.03	0.05	0.02	0.07	0.02	0.04	0.05	0.14	0.14	0.05	0.1 (0.0)	.32	.24

Incidence rates (IRs, per 100 000 inhabitants) are presented by year for bacterial, viral, and unspecified causes of meningitis and encephalitis. Means across the entire period and *P* values for linear trend analysis (2012–2019 and overall) are shown. Bold *P* values indicate statistical significance (*P* < .05). Patients with meningitis and encephalitis caused by unspecified pathogens are not shown. “Others” include *Mycobacterium tuberculosis, Treponema pallidum, Borrelia burgdorferi*, and leptospirosis (bacterial meningitis); adenovirus, measles virus, mumps virus, and other rare pathogens (viral meningitis); and measles virus, mumps virus, influenza virus, rabies virus, and mosquito-borne viruses (viral encephalitis). No “others” category was defined for bacterial encephalitis.

Abbreviations: CFR, case fatality rate; IR, incidence rate; overall, linear trend over the entire study period (2012–2021); *P* for trend ≤2019, linear trend analysis restricted to prepandemic years (2012–2019); SD, standard deviation; spp., species.

#### Meningitis

**Figure 1.**
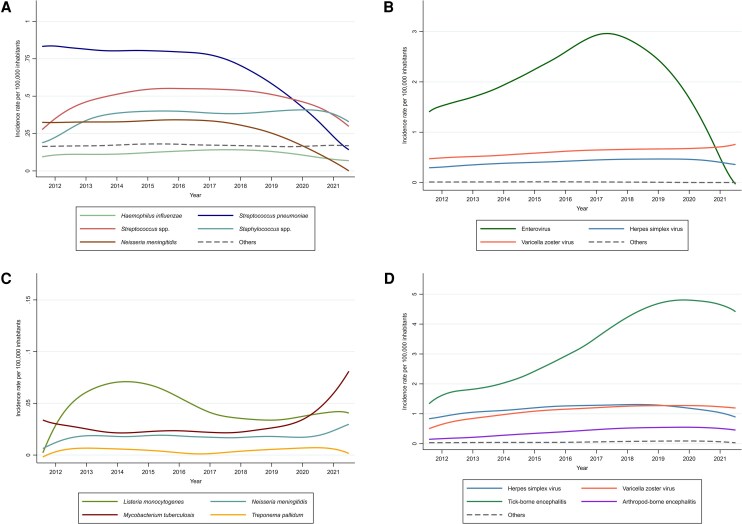
Temporal trends in pathogen-specific causes of meningitis and encephalitis. Incidence rates (IRs, per 100 000 inhabitants) per year for bacterial and viral pathogens in meningitis (*A* and *B*) and encephalitis (*C* and *D*) were calculated and visualized using LOWESS-smoothed curves. The displayed groups represent 80%–100% of defined pathogens. The analysis includes specified pathogens, while less common pathogens are grouped under “others” to illustrate overall trends for each condition. *A*, IRs of bacterial meningitis pathogens. “Others” include *Mycobacterium tuberculosis, Treponema pallidum, Borrelia burgdorferi*, and leptospirosis. *B*, IRs of viral meningitis pathogens. “Others” include adenovirus, measles virus, mumps virus, and other rare viral meningitis pathogens. *C*, IRs of bacterial encephalitis pathogens. No “others” category. *D*, IRs of viral encephalitis pathogens. “Others” include measles virus, mumps virus, influenza virus, rabies virus, and mosquito-borne viral encephalitis. Abbreviations: IR, incidence; LOWESS, locally weighted scatterplot smoothing; spp., species pluralis.

Overall incidence of bacterial meningitis remained stable over time, while pathogen-specific patterns varied. After 2019, *S. pneumoniae* and *Haemophilus influenzae* declined by more than 60% (Figure 1*A* and Table 2). Viral meningitis (56% of meningitis cases) increased modestly before 2019, followed by a sharp decline driven by a 90% reduction in enteroviral meningitis. In contrast, herpes simplex virus (HSV) and varicella-zoster virus (VZV) meningitis increased modestly over time (Figure 1*B* and Table 2).

#### Encephalitis

Bacterial encephalitis was rare (mean IR 1.5/100 000 inhabitants) but increased over time (Figure 1*C* and Table 2). Viral encephalitis, with a mean IR of 7.6/100 000 inhabitants, also increased, largely due to rising tick-born encephalitis (TBE) incidence. Arthropod-borne encephalitis showed a similar upward trend (Figure 1*D* and Table 2).

### Age-related Trends

#### Meningitis

**Figure 2.**
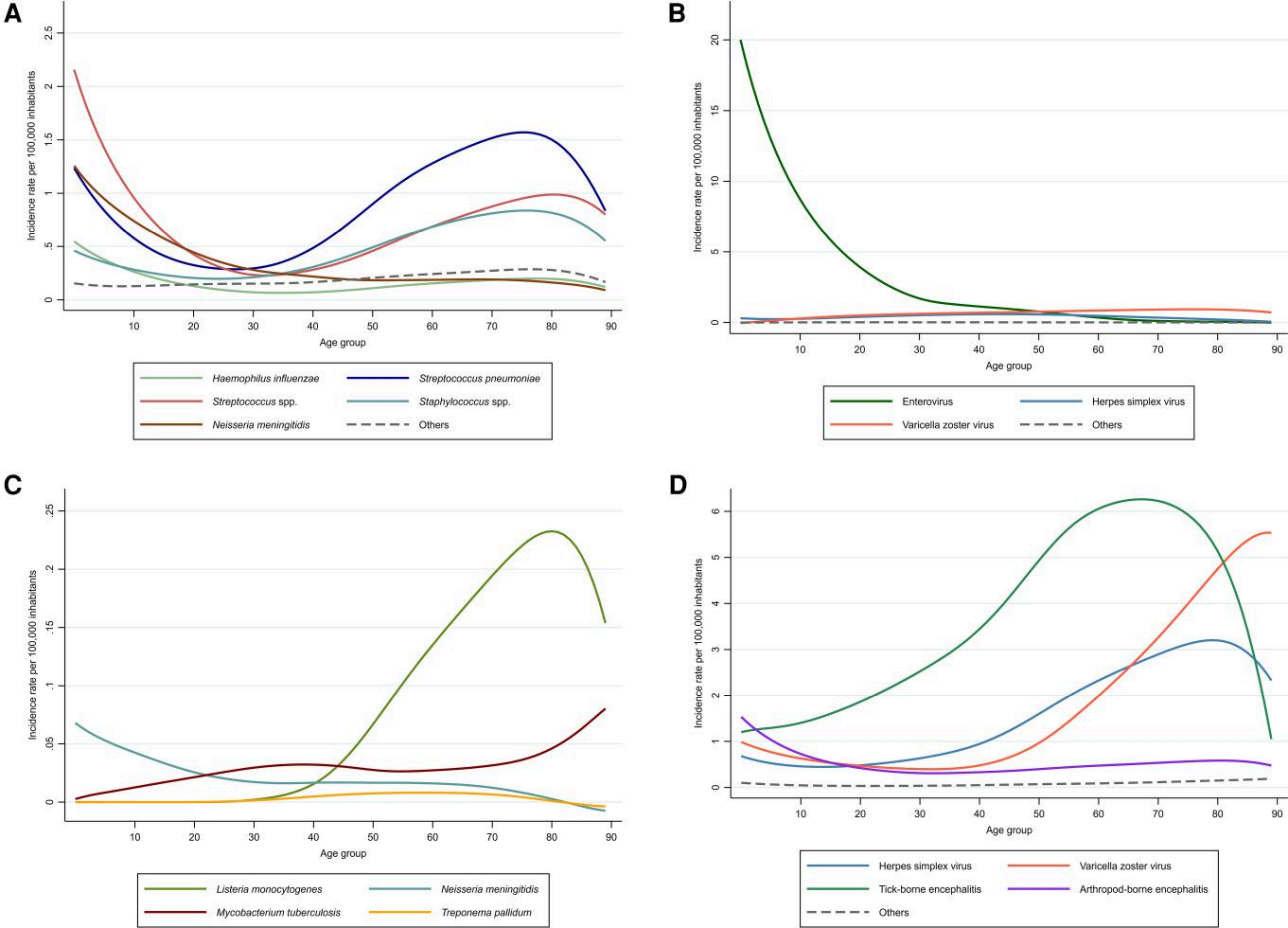
Patterns of pathogens across ages. Incidence rates (IRs, per 100 000 inhabitants) by age group for bacterial and viral pathogens in meningitis (*A* and *B*) and encephalitis (*C* and *D*) were calculated and visualized using LOWESS-smoothed curves (locally weighted scatterplot smoothing). The displayed groups represent 80%–100% of defined pathogens. Less common pathogens were grouped under “others,” analogous to Figure 1, to illustrate age-specific patterns. Unspecified pathogens are not shown in the graphs. *A*, IRs of bacterial meningitis pathogens by age group. “Others” include *Mycobacterium tuberculosis, Treponema pallidum, Borrelia burgdorferi*, and leptospirosis. *B*, IRs of viral meningitis pathogens by age group. “Others” include adenovirus, measles virus, mumps virus, and other rare viral meningitis pathogens. *C*, IRs of bacterial encephalitis pathogens by age group. No “Others” category. *D*, IRs of viral encephalitis pathogens by age group. “Others” include measles virus, mumps virus, influenza virus, rabies virus, and mosquito-borne viral encephalitis. Abbreviations: IR, incidence rate; LOWESS, locally weighted scatterplot smoothing; spp., species pluralis.

Bacterial meningitis exhibited a bimodal age distribution, with peaks among children younger than 10 years and adults 65 years of age or older. Several pathogens followed this pattern: *Streptococcus* species were most common in children younger than 5 years, whereas *S. pneumoniae* showed peaks in early childhood and again in older adults. *Neisseria meningitidis* was most frequent in young children and adolescents (Figure 2*A* and Table 3).

**Table 3.** Age-related Trends of Meningitis and Encephalitis

Diagnosis	Age Groups						
IR per 100 000 Inhabitants	0–10	11–20	21–49	50–65	>65	Mean (SD)	*P* for Trend
Overall	41.01	16.78	19.47	27.39	47.10	30.3 (13.3)	.33
Meningitis	31.27	9.51	9.28	9.00	13.14	14.4 (9.6)	.33
Bacterial meningitis	7.07	2.00	1.64	3.46	6.00	4.0 (2.4)	1.00
*Haemophilus influenzae*	0.40	0.09	0.05	0.09	0.18	0.2 (0.1)	.62
*Streptococcus pneumoniae*	0.90	0.19	0.28	0.89	1.53	0.8 (0.5)	.33
*Streptococcus* spp.	1.50	0.20	0.18	0.45	0.89	0.6 (0.6)	1.00
*Staphylococcus* spp.	0.39	0.15	0.20	0.51	0.78	0.4 (0.3)	.14
*Neisseria meningitidis*	0.97	0.55	0.15	0.19	0.17	0.4 (0.4)	.14
Others	0.17	0.06	0.17	0.12	0.31	0.2 (0.1)	.33
Viral meningitis	22.34	6.06	6.29	3.39	3.53	8.3 (8.0)	.14
Enterovirus	14.12	1.37	1.39	0.12	0.07	3.4 (6.0)	.**05**
Adenovirus	0.02	0.00	0.00	0.00	0.00	0.0 (0.0)	.16
Herpes simplex virus	0.30	0.13	0.60	0.45	0.24	0.3 (0.2)	1.00
Varicella-zoster virus	0.09	0.51	0.63	0.60	0.94	0.6 (0.3)	.**05**
Others	0.00	0.02	0.01	0.01	0.00	0.0 (0.0)	.80
Encephalitis	9.73	7.26	10.18	18.39	33.96	15.9 (10.9)	.**05**
Bacterial encephalitis	1.18	0.46	0.72	1.80	3.46	1.5 (1.2)	.14
*Listeria monocytogenes*	0.00	0.00	0.00	0.04	0.22	0.1 (0.1)	.**05**
*Neisseria meningitidis*	0.06	0.02	0.01	0.02	0.01	0.0 (0.0)	.**05**
*Mycobacterium tuberculosis*	0.01	0.02	0.03	0.02	0.03	0.0 (0.0)	.14
*Treponema pallidum*	0.00	0.00	0.00	0.02	0.00	0.0 (0.0)	.48
Viral encephalitis	5.57	3.25	5.01	8.15	16.43	7.7 (5.2)	.14
Herpes simplex virus	0.58	0.27	0.70	1.26	3.06	1.2 (1.1)	.**05**
Varicella-zoster virus	0.92	0.25	0.36	0.58	4.02	1.2 (1.6)	.33
Tick-borne	1.41	1.65	2.48	4.76	5.60	3.2 (1.9)	.**01**
Arthropod-borne	1.14	0.19	0.30	0.30	0.53	0.5 (0.4)	1.00
Others	0.07	0.01	0.04	0.06	0.13	0.1 (0.0)	.33

Incidence rates (IRs, per 100 000 inhabitants) are presented by age group for bacterial, viral, and unspecified causes of meningitis and encephalitis. Means across the entire period and *P* values for linear trend analysis by age. Bold *P* values indicate statistical significance (*P* < .05). Patients with meningitis and encephalitis caused by unspecified pathogens are not shown. “Others” include *Mycobacterium tuberculosis, Treponema pallidum, Borrelia burgdorferi*, and leptospirosis (bacterial meningitis); adenovirus, measles virus, mumps virus, and other rare pathogens (viral meningitis); and measles virus, mumps virus, influenza virus, rabies virus, and mosquito-borne viruses (viral encephalitis). No “others” category was defined for bacterial encephalitis.

Abbreviations: CFR, case fatality rate; IR, incidence rate (per 100 000 inhabitants); *P* for trend: linear trend across all age groups (0–99 years); SD, standard deviation; spp., species.

Viral meningitis was most common in children younger than 10 years, primarily caused by enteroviruses, and declined with age. In contrast, VZV meningitis increased with age. Notably, more than half of viral meningitis cases lacked a specified pathogen (Figure 2*B* and Table 3).

#### Encephalitis

Bacterial encephalitis remained rare across all age groups, with increasing incidence up to 80 years of age. More than 90% of encephalitis cases were pathogen unspecified. *L. monocytogenes* was the most frequently identified bacterial cause, especially among adults above 65 years (Figure 2*C* and Table 3).

Viral encephalitis incidence exceeded bacterial incidence across all age groups. HSV-, VZV-, and TBE-related encephalitis increased with age (Figure 2*D* and Table 3).

### Clinical Outcomes

**Figure 3.**
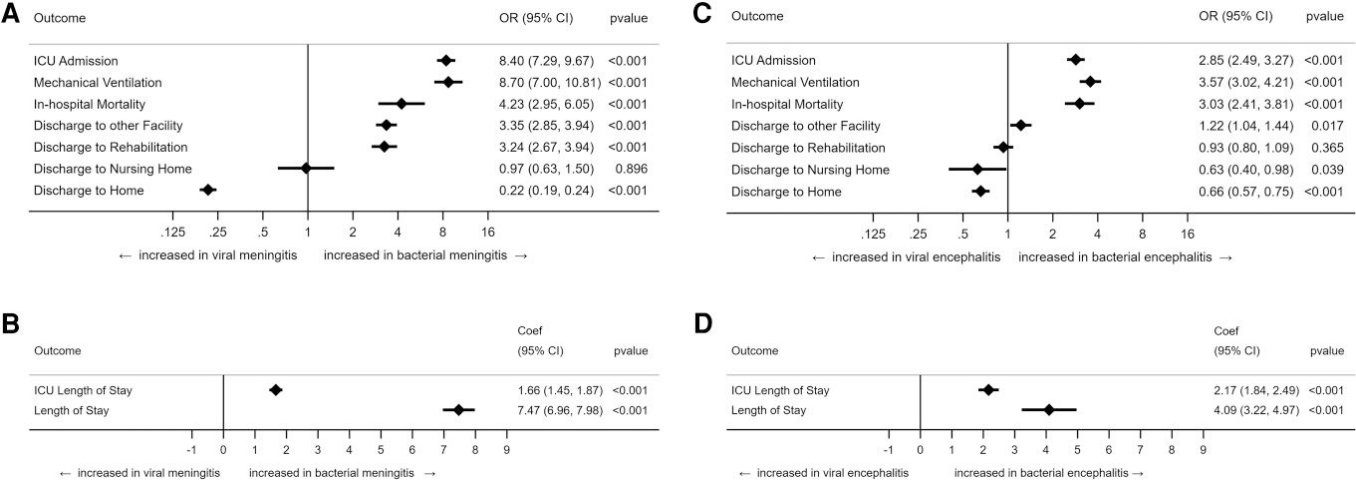
Clinical outcomes in bacterial versus viral meningitis and encephalitis. Odds ratios (OR), coefficients, and 95% confidence intervals (CI) for clinical outcomes adjusted for age, sex, and comorbidities. Discharge destinations include transfer to another postacute care facility, rehabilitation, nursing home, or home. *A*, Odds ratios (OR) and 95% CI for adverse outcomes in bacterial versus viral meningitis. *B*, Coefficients and 95% CI for continuous outcomes: length of stay (LOS) in hospital and intensive care unit (ICU) in bacterial versus viral meningitis. *C*, Adverse outcomes in bacterial versus viral encephalitis. *D*, Length of stay (LOS) in hospital and ICU in bacterial versus viral encephalitis. Abbreviations: CI, confidence interval; ICU, intensive care unit; LOS, length of stay; OR, odds ratio.

Bacterial CNS infections were associated with consistently worse clinical outcomes compared with viral infections (Figure 3; Supplementary Table 3).

#### Meningitis

In-hospital mortality was 6.9% for bacterial meningitis and 0.7% for viral meningitis. Bacterial meningitis was associated with substantially higher odds of ICU admission and mechanical ventilation, longer hospital stays, and less frequent discharge home, with more transfers to rehabilitation or other long-term care facilities (Figure 3*A* and *B*).

#### Encephalitis

In-hospital mortality was 12.9% for bacterial encephalitis and 3.6% for viral encephalitis. Bacterial encephalitis was associated with higher ICU utilization, longer hospital stays, and fewer discharges home, with more transfers to rehabilitation or nursing facilities (Figure 3*C* and *D*).

#### Case Fatality Rates

**Figure 4.**
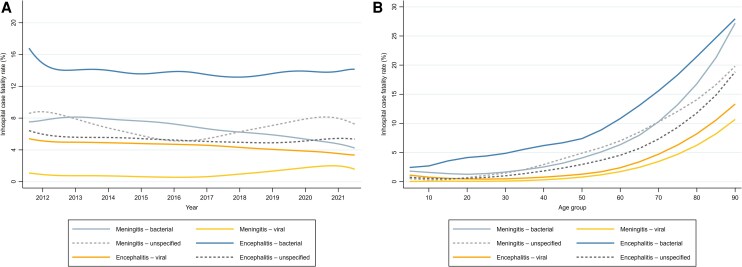
Case fatality rates in meningitis and encephalitis. Case fatality rates (CFRs), defined as in-hospital mortality in percent, were visualized using LOWESS-smoothed curves. Pathogens were categorized as bacterial, viral, or unspecified. *A*, Temporal trends in CFRs for meningitis and encephalitis by pathogen category. *B*, Age-specific CFRs for meningitis and encephalitis by pathogen category. Abbreviations: CFR, case fatality rate; LOWESS, locally weighted scatterplot smoothing.

For bacterial meningitis, mean CFR was 6.9% and declined slightly over time, whereas mean CFR for viral meningitis remained low (0.9%) without a changing temporal trend. For bacterial encephalitis, mean CFR was 13.0% and remained stable over time, while mean CFR in viral encephalitis was 3.7% and declined modestly (Figure 4*A*; Supplementary Table 4).

Across all CNS infection types, CFR increased markedly with age. Among patients 80 years or older, CFR exceeded 20% for bacterial CNS infections, whereas CFR from viral infections, although age-dependent, remained substantially lower (Figure 4*B*; Supplementary Table 5).

## DISCUSSION

In this nationwide cohort study of hospitalized patients with infectious meningitis and encephalitis, 3 main findings emerged. First, hospitalization rates for CNS infections increased between 2012 and 2019 and declined markedly in 2020–2021, with the sharpest declines seen for enteroviral and other droplet-transmitted pathogens (eg, *N. meningitidis*, *S. pneumoniae*, and *H. influenzae*), whereas incidence for TBE continued to rise. Second, pathogen distribution varied substantially by age, with enteroviruses and *Streptococcus* spp. dominating in children, *N. meningitidis* in adolescents, and *S. pneumoniae*, *L. monocytogenes*, TBE virus, and VZV in older adults. Third, bacterial CNS infections were consistently associated with worse in-hospital outcomes than viral infections, particularly among older patients. Although derived from Swiss nationwide data, these patterns are likely relevant to other high-income countries with comparable healthcare systems and vaccination programs, having implications for vaccination policy, clinical risk stratification, and surveillance.

Compared with prior studies that focused on selected pathogens and age groups, this analysis includes both meningitis and encephalitis across all ages over a decade. The prepandemic increase in incidence was driven by viral pathogens, followed by marked declines in 2020–2021. Bacterial meningitis incidence remained through 2019, and then declined for *S. pneumoniae*, *H. influenzae*, and *N. meningitidis*, consistent with reports from other high-income countries [8, 13]. Viral meningitis exhibited greater temporal variability; enteroviral meningitis declined by approximately 90% in 2020. These effects are in line with global observations and likely reflect the dual effect of reduced pathogen circulation due to COVID-19-related nonpharmaceutical interventions (especially wearing face masks) and reduced healthcare utilization, particularly among patients with milder viral diseases [1, 9, 14–18]. In contrast, TBE incidence increased during and before the pandemic in Switzerland and other European countries, possibly due to behavioral shifts toward outdoor activities [19], suboptimal vaccination coverage in endemic regions, and expanding tick habitats linked to climate change [20–22].

Age-specific patterns differed by pathogen. Bacterial meningitis showed a bimodal distribution, with peaks in young children and older adults, consistent with global epidemiology. *S. pneumoniae* remained the leading bacterial pathogen, particularly among young children and older adults, whereas *N. meningitidis* predominated in children and adolescents [4–8]. These patterns mirror current Swiss vaccination recommendations for pneumococcal and meningococcal disease by the Federal Commission for Vaccination Issues [23]. Viral meningitis occurred predominantly in children, driven by enteroviruses [9, 15, 24, 25]. Encephalitis, by contrast, primarily affected older adults, with increasing incidence of TBE virus, HSV, and VZV. HSV and VZV encephalitis were especially frequent among older adults, consistent with international data and likely reflecting the consequence of immunosenescence and a higher prevalence of immunosuppression in this age group [25, 26]. *L. monocytogenes* also increased with age, supporting current guideline recommendations for empirical coverage in at-risk populations, including age [2, 4]. In Switzerland, TBE emerged as a leading cause of viral encephalitis, with incidence peaking in older adults, differing from patterns reported in some neighboring countries and likely reflecting regional differences in vaccination uptake, exposure, and ecology [20–22, 26]. In a survey in Switzerland in 2018, only 33% had a complete vaccination (3 doses) for TBE, and only 10% received an indicated booster vaccination [22].

Bacterial CNS infections were associated with substantially worse outcomes than viral CNS infections, including higher rates of ICU admission, mechanical ventilation, longer LOS, and higher in-hospital mortality [5, 27]. In-hospital mortality for bacterial meningitis was nearly 7%, still somewhat lower than the 10%–15% reported in other high-income settings, possibly reflecting differences in case ascertainment, reliance on in-hospital mortality, and access to rapid diagnostics and intensive care [1, 8, 26–28]. Mortality for viral encephalitis was consistent with prior reports (3.6%), although bacterial encephalitis carried a higher risk (12.9%) [27, 29]. In our cohort, mortality for bacterial meningitis and viral encephalitis declined modestly over time. Improvements in diagnostics with the availability of multiplex polymerase chain reaction (PCR), where results are available within 2 to 3 hours, but also in supportive care, antimicrobial therapy, and vaccine availability have contributed to declining mortality from bacterial and viral CNS infections over recent decades [3, 28, 30]. However, mortality increased sharply with age, exceeding 20% among patients 80 years of age or older, underscoring the vulnerability of this population and the need to optimize vaccination efforts. Beyond mortality, CNS infections were associated with substantial morbidity, including frequent discharge to rehabilitation or long-term care [5, 8, 27]. Not only bacterial meningitis, but also viral CNS infections can result in persistent symptoms and functional impairment, particularly after HSV- and VZV-related encephalitis [9, 29, 31].

Vaccination remains central in the prevention of CNS infections [21, 30, 32]. To date, not only vaccinations against meningococci, *H. influenzae* type b, VZV and TBE are available, but also pneumococcal conjugate vaccines (PCV), which are regularly adapted to the circulating pneumococcal serotypes. As a consequence, the incidence of invasive pneumococcal disease, including bacterial meningitis, was significantly reduced in many settings, although the high IR in older patients in high-income countries suggests persistent gaps in adult vaccination, vaccine hesitancy, and missed opportunities for immunization [4, 8, 13, 33, 34]. In a survey in Switzerland in 2022, only 9.6% of people older than 65 years of age was vaccinated as recommended by national guidelines [35]. Additionally, an aging population and immunosenescence—often compounded by comorbidities and immunosuppressive conditions—may further limit vaccine effectiveness at the population level [2, 4, 28, 33]. These challenges and the observed epidemiological patterns are regularly addressed by Swiss vaccination policy [23]. For pneumococci a polyvalent conjugate vaccine is recommended (all children, adults ≥ 65 years, younger adults if at risk for invasive disease), which to date is PCV21 or PCV20. Patients vaccinated with a lower valent conjugate vaccine (PCV13 or PCV15) or even a polysaccharide vaccine should receive a booster vaccine, to cover a broader spectrum of pneumococcal serotypes and to optimize vaccine effectiveness by reducing also colonization rate. This represents one of the most effective measures to reduce disease burden [35]. Similarly, the identification of *N. meningitidis* as the predominant pathogen in adolescents underscores the importance of meningococcus-specific prevention. Vaccination against meningococcal serotypes ACWY and B is recommended in all children [23, 36, 37]. Likewise, the recombinant herpes zoster vaccine (Shingrix®), introduced in Switzerland in 2021, offers effective protection against VZV reactivation and its complications in older adults and immunocompromised hosts and is recommended in adults ≥65 years and younger adults if immunocompromised [23, 36]. Tick-borne encephalitis vaccination is recommended in endemic areas, which meanwhile includes most of Switzerland [20–22]. As discussed above, due to expanding tick habitats linked to climate change and a suboptimal vaccination uptake, there is a persistent rise in TBE cases [22], which underscores the need for improved public awareness in endemic regions to optimize vaccination rate [20, 22, 38].

An important challenge in our study was the high proportion of unspecified pathogens, particularly in encephalitis, consistent with prior research [1, 5, 25, 39]. Notably, over 90% of infectious encephalitis cases lacked specified etiology, which substantially limits the pathogen-specific interpretability of this subgroup. Although the multiplex PCR panel (BIOFIRE® FILMARRAY® Meningitis/Encephalitis Panel) was increasingly used in Swiss hospitals from around 2016 onward, the proportion of CNS infections coded without a specified pathogen did not substantially decline over time. This suggests that the persistently high proportion of unspecified etiologies likely reflects limitations of administrative ICD-10-GM coding more than diagnostic test availability alone [40, 41]. Emerging technologies such as metagenomic next-generation sequencing may improve diagnostic yield but remain limited by cost and implementation barriers [42]. Consequently, improving outcomes in infectious meningitis and encephalitis will require coordinated efforts across 4 key domains: closing vaccination gaps, ensuring timely and equitable access to diagnostic evaluation, strengthening pathogen identification through optimized integration of clinical, administrative, serological, microbiological, and surveillance data, including further development of currently available multiplex PCR tests, and optimizing clinical management and antimicrobial treatment.

Key strengths of this study include nationwide coverage, a decade-long observation period, inclusion of all age groups and major CNS infection types (viral and bacterial meningitis and encephalitis).

Several limitations must be acknowledged in the context of the study design. First, the retrospective design and reliance on administrative data introduce potential misclassification and underreporting of specific pathogens. Furthermore, there is a lack of detailed clinical and laboratory information, absence of vaccination status, and postdischarge outcomes, underestimating the true disease burden. In particular, the high proportion of unspecified viral meningitis (>50%) and bacterial encephalitis (>90%) cases reflect limited etiological resolution, constraining pathogen-specific analyses and potentially obscuring smaller outbreak-related or rare diagnoses. Accordingly, rare pathogen-specific signals should be interpreted cautiously, as small absolute case numbers, reliance on ICD-based ascertainment, and smoothed nationwide incidence curves may obscure short-term outbreaks or exaggerate year-to-year variation. Second, the delay between symptom onset and adequate antimicrobial therapy—an important prognostic factor, particularly in bacterial meningitis—could not be assessed. Third, the lack of linkage with microbiological or vaccination data may also affect the validity and comprehensiveness of our findings. Fourth, clinical outcomes were analyzed at the level of bacterial versus viral infection categories. This deliberate choice preserves the epidemiological focus and statistical robustness of the analysis across broad temporal and demographic scales. Pathogen-specific outcome data would provide greater clinical granularity and should be addressed in future dedicated studies. Fifth, the Jonckheere-Terpstra test across the full study period (2012–2021) should be interpreted cautiously, as the sharp pandemic-related decline may violate the assumption of a monotonic trend; the prepandemic analysis (2012–2019) likely provides more robust trend estimates. Finally, generalizability beyond Switzerland may be constrained by differences in public health recommendations, health systems and epidemiology.

## CONCLUSION

This nationwide analysis demonstrates distinct temporal and age-related patterns of CNS infections in Switzerland and highlights the substantial burden and severity of mainly bacterial CNS infections, particularly among older adults. Pandemic-related public health measures during COVID-19 were associated with transient declines in several droplet-transmitted pathogens (eg, enteroviruses), whereas TBE incidence continued to rise, despite vaccine availability. These findings have important implications for clinical care and public health in Switzerland and comparable high-income settings. Decreasing incidence and improving outcomes in CNS infections will require targeted, age- and risk-adapted vaccination strategies, including vaccinations against pneumococci, meningococci, TBE, VZV, and *H. influenzae* type b, but also early recognition, timely microbiological diagnostics and optimized clinical management including rapid start of an empiric antimicrobial therapy. Assessment of the causing pathogen should be a key target through broader use of molecular diagnostics and closer integration of clinical, administrative, serological, microbiological, and surveillance data.

## Supplementary Material

ofag350_Supplementary_Data

## Supplementary Data

Supplementary materials are available at *Open Forum Infectious Diseases* online. Consisting of data provided by the authors to benefit the reader, the posted materials are not copyedited and are the sole responsibility of the authors, so questions or comments should be addressed to the corresponding author.
