# Supplementary material for: Nationwide Trends in Hospitalizations and Clinical Outcomes for Meningitis and Encephalitis: A 10-year Swiss Population-based Study
Source: Open Forum Infect Dis. 2026 Jun 5;13(6):ofag350. doi: 10.1093/ofid/ofag350 (PMC13263528; doi:10.1093/ofid/ofag350)
Supplement: ofag350_Supplementary_Data [file ofag350_supplementary_data.zip › Supplementary Online Material_OFID_clean.docx]

**Supplementary Appendix**

This appendix has been provided by the authors to give readers additional information about their work.

Supplement to: Essmann L. et al., Nationwide trends in hospitalizations and clinical outcomes for meningitis and encephalitis: A 10-year Swiss population-based study

Table of contents

Contents

[eTable 1 – ICD-10 GM codes for meningitis and encephalitis 3](#_Toc229646904)

[eTable 2 - Baseline characteristics of patients with viral, bacterial, and unspecified encephalitis and meningitis 4](#_Toc229646905)

[eTable 3: Outcomes in viral and bacterial meningitis and encephalitis 5](#_Toc229646906)

[eTable 4: Case fatality rate (CFR) in % by year for meningitis and encephalitis 6](#_Toc229646907)

[eTable 5: Case fatality rate (CFR) in % by age group for meningitis and encephalitis 7](#_Toc229646908)

[eFigure 1: Flowchart of hospitalizations for meningitis and encephalitis (2012 - 2021) 8](#_Toc229646909)

[eFigure 2: Pathogen categories over time and across age groups 8](#_Toc229646910)

# eTable 1 – ICD-10 GM codes for meningitis and encephalitis

| **Category** | **Pathogen** | **ICD-Code** |
| --- | --- | --- |
| **Bacterial meningitis** | *Haemophilus influenzae* | **G00.0** |
|  | *Streptococcus pneumoniae* | **G00.1** |
|  | *Streptococcus* spp*.* | **G00.2** |
|  | *Staphylococcus* spp*.* | **G00.3** |
|  | *Listeria monocytogenes* | **A32.1 + G01** |
|  | *Neisseria meningitidis* | **A39.0** |
|  | *Mycobacterium tuberculosis* | **A17.0** |
|  | *Neisseria gonorrhoeae* | **A54.8 + G01** |
|  | *Treponema pallidum* | **A52.1, A51.4, A50.4 + G01** |
|  | *Borrelia burgdorferi* | **A69.2 + G01** |
|  | *Salmonella* spp. | **A02.2 + G01** |
|  | *Salmonella typhi* | **A01 + G01** |
|  | *Yersinia pestis* | **A20.3** |
|  | *Bacillus anthracis* | **G01 + A22.8** |
|  | *Leptospira* spp. | **A27.8, A27.9 + G01** |
|  | Other specified bacterial meningitis | **G00.8, G00.9** |
| **Viral meningitis** | Enterovirus | **A87.0** |
|  | Adenovirus | **A87.1** |
|  | Herpes simplex virus | **B00.3** |
|  | Measles virus | **B05.1** |
|  | Mumps virus | **B26.1** |
|  | Rubella virus | **B06.0 + G02, G03** |
|  | Varicella zoster virus | **B02.1, B01.0** |
|  | Unspecified viral meningitis | **G02, G03, A87.8, A87.9** |
| **Bacterial encephalitis** | *Listeria monocytogenes* | **A32.1 + G05.0** |
|  | *Neisseria meningitidis* | **A39 + G05.0** |
|  | *Mycobacterium tuberculosis* | **A17.8 + G05.0** |
|  | *Treponema pallidum* | **A52.1, A51.4, A50.4 + G05.0** |
|  | Bacterial meningoencephalitis | **G04.2** |
| **Viral encephalitis** | Enterovirus | **A85.0** |
|  | Herpes simplex virus | **B00.4** |
|  | Varicella zoster virus | **B01.1, B02.0** |
|  | Measles virus | **B05.0** |
|  | Mumps virus | **B26.2** |
|  | Rubella virus | **B06.0 + G05.1** |
|  | Tick-borne encephalitis virus | **A84** |
|  | Arthropod-borne viruses | **A85** |
|  | Mosquito-borne viruses | **A83** |
|  | Rabies virus | **A82** |
|  | Influenza virus | **J10.8, J11.8, J0 + G05.1** |
|  | Unspecified viral encephalitis | **A86** |
| **Unspecified causes** | Unspecified encephalitis | **G04.8, G04.9** |

**Abbreviations**: ICD = International Classification of Diseases; spp.= species pluralis

# eTable 2 - ****Baseline characteristics of patients with viral, bacterial, and unspecified encephalitis and meningitis****

|  | **Bacterial meningitis** | **Viral meningitis** | **Unspecified meningitis** | **Bacterial encephalitis** | **Viral encephalitis** | **Unspecified encephalitis** |
| --- | --- | --- | --- | --- | --- | --- |
|  | **N=2,840** | **N=5,658** | **N=1,662** | **N=1,223** | **N=6,368** | **N=5,675** |
| **Age, years, mean (SD)** | 45.0 (28.7) | 27.9 (24.1) | 49.2 (24.9) | 55.9 (23.5) | 53.5 (23.6) | 53.9 (21.8) |
| **Age categories, n (%)** |  |  |  |  |  |  |
| **0-10 years** | 598 (21.1) | 1,889 (33.4) | 158 (9.5) | 100 (8.2) | 471 (7.4) | 252 (4.4) |
| **11-20 years** | 171 (6.0) | 518 (9.2) | 124 (7.5) | 39 (3.2) | 278 (4.4) | 304 (5.4) |
| **21-49 years** | 554 (19.5) | 2,124 (37.5) | 454 (27.3) | 242 (19.8) | 1,690 (26.5) | 1,505 (26.5) |
| **50-65 years** | 597 (21.0) | 586 (10.4) | 371 (22.3) | 311 (25.4) | 1,408 (22.1) | 1,457 (25.7) |
| **>65 years** | 920 (32.4) | 541 (9.6) | 555 (33.4) | 531 (43.4) | 2,521 (39.6) | 2,157 (38.0) |
| **Male, n (%)** | 1,563 (55.0) | 3,019 (53.4) | 922 (55.5) | 670 (54.8) | 3,716 (58.4) | 2,900 (51.1) |
| **Elixhauser comorbidity index, mean (SD)** | 1.8 (1.9) | 0.5 (1.1) | 1.8 (2.0) | 2.7 (2.2) | 1.6 (1.8) | 2.0 (1.9) |
| **In-hospital frailty score categories, n (%)** |  |  |  |  |  |  |
| **< 5 points** | 1,786 (62.9) | 5,272 (93.2) | 1,170 (70.4) | 493 (40.3) | 4,354 (68.4) | 3,596 (63.4) |
| **5-15 points** | 904 (31.8) | 345 (6.1) | 418 (25.2) | 608 (49.7) | 1,781 (28.0) | 1,792 (31.6) |
| **> 15 points** | 150 (5.3) | 41 (0.7) | 74 (4.5) | 122 (10.0) | 233 (3.7) | 287 (5.1) |
| **Comorbidities, n (%)** |  |  |  |  |  |  |
| **Obesity** | 44 (1.5) | 33 (0.6) | 29 (1.7) | 25 (2.0) | 53 (0.8) | 101 (1.8) |
| **Diabetes mellitus** | 278 (9.8) | 172 (3.0) | 157 (9.4) | 183 (15.0) | 561 (8.8) | 697 (12.3) |
| **Atherosclerotic cardiovascular diseaseᵃ** | 531 (18.7) | 186 (3.3) | 297 (17.9) | 334 (27.3) | 749 (11.8) | 1,053 (18.6) |
| **Chronic pulmonary diseaseᵇ** | 105 (3.7) | 115 (2.0) | 92 (5.5) | 81 (6.6) | 358 (5.6) | 409 (7.2) |
| **URTI or ENT infectionsᶜ** | 396 (13.9) | 225 (4.0) | 120 (7.2) | 183 (15.0) | 135 (2.1) | 145 (2.6) |
| **LRTIᵈ** | 241 (8.5) | 152 (2.7) | 123 (7.4) | 138 (11.3) | 274 (4.3) | 314 (5.5) |
| **Malignancyᵉ** | 428 (15.1) | 303 (5.4) | 349 (21.0) | 176 (14.4) | 405 (6.4) | 731 (12.9) |
| **Immunodeficiencyᶠ** | 88 (3.1) | 124 (2.2) | 60 (3.6) | 76 (6.2) | 221 (3.5) | 218 (3.8) |
| **HIV infection** | 25 (0.9) | 111 (2.0) | 10 (0.6) | 15 (1.2) | 45 (0.7) | 42 (0.7) |
| **Splenectomy** | 23 (0.8) | 2 (0.0) | 1 (0.1) | 1 (0.1) | 2 (0.0) | 1 (0.0) |
| **Alcohol dependence** | 108 (3.8) | 55 (1.0) | 60 (3.6) | 90 (7.4) | 159 (2.5) | 185 (3.3) |
| **Liver disease, including cirrhosis** | 20 (0.7) | 4 (0.1) | 11 (0.7) | 22 (1.8) | 17 (0.3) | 32 (0.6) |
| **Chronic kidney disease** | 200 (7.0) | 150 (2.7) | 113 (6.8) | 162 (13.2) | 577 (9.1) | 459 (8.1) |
| **Craniofacial anomalies** | 1 (0.0) | 3 (0.1) | 1 (0.1) | 0 (0.0) | 0 (0.0) | 4 (0.1) |
| **Cerebrospinal fluid fistula** | 83 (2.9) | 14 (0.2) | 33 (2.0) | 21 (1.7) | 1 (0.0) | 26 (0.5) |
| **Presence of cochlear implant** | 6 (0.2) | 0 (0.0) | 1 (0.1) | 2 (0.2) | 3 (0.0) | 0 (0.0) |

**Abbreviations**: SD: standard deviation; HIV: human immunodeficiency virus; ENT - Ear, nose, and throat; URTI – Upper respiratory tract infection, LRTI – Lower respiratory tract infection

ᵃ ASCVD (atherosclerotic cardiovascular disease) includes coronary artery disease, peripheral arterial disease, and cerebrovascular disease.

ᵇ Chronic pulmonary disease includes chronic obstructive pulmonary disease (COPD), asthma, and obstructive sleep apnoea syndrome (OSAS).

ᶜ URTI and ENT infections include external and middle ear infections (e.g., otitis media, mastoiditis) and acute upper respiratory infections (e.g., sinusitis, pharyngitis).

ᵈ LRTI includes influenza, pneumonia, and bronchitis (ICD-10 J09–J22).

ᵉ Malignancy includes both hematologic cancers and solid tumors.

ᶠ Immunodeficiency was defined by ICD-10 codes: primary immunodeficiencies (D80–D84), agranulocytosis (D70), immunodeficiency after chemotherapy or immunosuppressants (D90), other immune disorders (D89), transplant status (Z94), and other specified immunodeficiencies (D84.8).

# eTable 3: Outcomes in viral and bacterial meningitis and encephalitis

|  | **Bacterial meningitis** | **Viral meningitis** | **Effect measure*** | **p-value** | **Bacterial encephalitis** | **Viral encephalitis** | **Effect measure*** | **p-value** |
| --- | --- | --- | --- | --- | --- | --- | --- | --- |
|  | **N=2,840** | **N=5,658** |  |  | **N=1,223** | **N=6,368** |  |  |
| **Mortality, n (%)** | 196 (6.9) | 40 (0.7) | 4.23 (2.95-6.05) | <0.001 | 158 (12.9) | 227 (3.6) | 3.03 (2.41-3.81) | <0.001 |
| **ICU admission, n (%)** | 1,165 (41.0) | 343 (6.1) | 8.40 (7.29-9.67) | <0.001 | 566 (46.3) | 1,219 (19.1) | 2.85 (2.49-3.27) | <0.001 |
| **Mechanical ventilation, n (%)** | 588 (20.7) | 113 (2.0) | 8.70 (7.00-10.81) | <0.001 | 357 (29.2) | 493 (7.7) | 3.57 (3.02-4.21) | <0.001 |
| **ICU LOS (days), mean (SD)** | 2.6 (7.1) | 0.3 (2.1) | 1.66 (1.45-1.87) | <0.001 | 4.1 (9.1) | 1.1 (4.3) | 2.17 (1.84-2.49) | <0.001 |
| **General LOS (days), mean (SD)** | 17.5 (15.6) | 6.2 (8.1) | 7.47 (6.96-7.98) | <0.001 | 20.7 (17.6) | 14.5 (13.9) | 4.09 (3.22-4.97) | <0.001 |
| **Discharge to rehabilitation, n (%)** | 488 (17.2) | 170 (3.0) | 3.35 (2.85-3.94) | <0.001 | 284 (23.2) | 1,339 (21.0) | 1.22 (1.04-1.44) | 0.017 |
| **Facility discharge, n (%)** | 570 (20.1) | 285 (5.0) | 3.24 (2.67-3.94) | <0.001 | 234 (19.1) | 904 (14.2) | 0.93 (0.80-1.09) | 0.365 |
| **Discharge to nursing home, n (%)** | 59 (2.1) | 37 (0.7) | 0.97 (0.63-1.50) | 0.896 | 25 (2.0) | 162 (2.5) | 0.63 (0.40-0.98) | 0.039 |
| **Discharge home, n (%)** | 1,540 (54.2) | 5,132 (90.7) | 0.22 (0.19-0.24) | <0.001 | 521 (42.6) | 3,817 (59.9) | 0.66 (0.57-0.75) | <0.001 |

***Effect measure**: Odds ratios (ORs) with 95% confidence intervals (CIs) for binary outcomes (e.g., in-hospital mortality, ICU admission, mechanical ventilation, and discharge disposition). Adjusted mean differences with 95% CIs for continuous outcomes (e.g., ICU LOS, LOS)

**Abbreviations: ICU = intensive care unit; LOS = length of stay; SD = standard deviation, OR = odds ratio; CI= confidence interval**

| **Diagnosis** | **Years** |  |  |  |  |  |  |  |  |  |  | **P for trend** | |
| --- | --- | --- | --- | --- | --- | --- | --- | --- | --- | --- | --- | --- | --- |
| **CFR in %** | **2012** | **2013** | **2014** | **2015** | **2016** | **2017** | **2018** | **2019** | **2020** | **2021** | **Mean (SD)** | **≤2019** | **overall** |
| **Overall** | 5.20 | 3.61 | 4.88 | 5.19 | 4.23 | 4.16 | 3.35 | 3.78 | 5.78 | 3.95 | 4.4 (0.8) | 0.08 | 0.42 |
| **Meningitis** | 4.27 | 2.85 | 4.18 | 3.70 | 2.39 | 3.38 | 1.86 | 3.23 | 5.90 | 3.22 | 3.5 (1.1) | 0.08 | 0.42 |
| **Bacterial meningitis** | 8.89 | 6.43 | 8.61 | 8.50 | 5.05 | 8.48 | 6.05 | 5.26 | 7.29 | 4.13 | 6.9 (1.7) | **0.05** | **0.03** |
| **Viral Meningitis** | 0.97 | 0.30 | 0.40 | 1.06 | 0.34 | 0.43 | - | 0.81 | 4.05 | 0.29 | 0.9 (1.2) | 0.80 | 0.93 |
| **Encephalitis** | 6.16 | 4.41 | 5.55 | 6.60 | 5.68 | 4.81 | 4.46 | 4.22 | 5.72 | 4.30 | 5.2 (0.9) | 0.14 | 0.18 |
| **Bacterial encephalitis** | 11.59 | 11.39 | 12.50 | 19.64 | 12.03 | 7.58 | 17.78 | 9.47 | 17.56 | 10.56 | 13.0 (4.0) | 0.80 | 0.79 |
| **Viral Encephalitis** | 4.62 | 3.43 | 4.14 | 4.85 | 3.34 | 4.34 | 2.88 | 3.28 | 3.45 | 2.53 | 3.7 (0.8) | 0.14 | 0.06 |

# ****eTable 4: Case fatality rate (CFR) in % by year for meningitis and encephalitis****

**Abbreviations**: CFR = Case Fatality Rate; SD = Standard Deviation

# eTable 5: Case fatality rate (CFR) in % by age group for meningitis and encephalitis

| **Diagnosis** | **Age groups** | | |  | |  | |  | |  | |  | |  | |  | |  | |  | |  | |  | |  | |  | |  | |  | |  | |  | |  | |  |
| --- | --- | --- | --- | --- | --- | --- | --- | --- | --- | --- | --- | --- | --- | --- | --- | --- | --- | --- | --- | --- | --- | --- | --- | --- | --- | --- | --- | --- | --- | --- | --- | --- | --- | --- | --- | --- | --- | --- | --- | --- |
| **CFR in %** | **0-4** | **5-9** | **10-14** | | **15-19** | | **20-24** | | **25-29** | | **30-34** | | **35-39** | | **40-44** | | **45-49** | | **50-54** | | **55-59** | | **60-64** | | **65-69** | | **70-74** | | **75-79** | | **80-84** | | **85-89** | | **90-94** | | **95-99** | | **Mean (SD)** | **P for trend** |
| **Overall** | 0.99 | 0.12 | - | | 0.64 | | 0.63 | | 0.50 | | 0.69 | | 1.00 | | 1.48 | | 2.26 | | 3.33 | | 3.67 | | 4.70 | | 6.34 | | 6.94 | | 10.90 | | 12.88 | | 17.22 | | 27.37 | | 17.24 | | 7.2 (9.4) | **< 0.01** |
| **Meningitis** | 0.68 | 0.19 | - | | 0.93 | | - | | 0.37 | | 0.32 | | 0.76 | | 1.63 | | 1.26 | | 3.81 | | 4.53 | | 5.61 | | 7.80 | | 6.36 | | 13.73 | | 12.77 | | 21.71 | | 28.07 | | 42.86 | | 7.7 (1.3) | **< 0.01** |
| **Bacterial meningitis** | 2.39 | 1.20 | - | | 3.03 | | - | | 1.35 | | - | | 2.13 | | 6.19 | | 0.74 | | 6.90 | | 3.21 | | 8.47 | | 8.86 | | 8.33 | | 15.69 | | 16.42 | | 28.26 | | 44.44 | | 42.86 | | 10.0 (13.5) | **< 0.01** |
| **Viral Meningitis** | 0.07 | - | - | | 0.37 | | - | | - | | - | | - | | - | | 0.85 | | 0.81 | | 2.11 | | 0.67 | | 3.82 | | 2.07 | | 7.14 | | 4.76 | | 12.00 | | 15.79 | | - | | 2.7 (4.5) | **< 0.01** |
| **Encephalitis** | 2.31 | - | - | | 0.28 | | 1.42 | | 0.65 | | 1.12 | | 1.24 | | 1.37 | | 2.83 | | 3.05 | | 3.29 | | 4.27 | | 5.71 | | 7.16 | | 9.92 | | 12.92 | | 15.23 | | 27.05 | | 9.09 | | 6.8 (8.9) | **< 0.01** |
| **Bacterial encephalitis** | 6.45 | - | - | | - | | 15.00 | | 3.33 | | 7.41 | | 2.86 | | 2.08 | | 9.30 | | 8.99 | | 7.44 | | 11.88 | | 14.29 | | 22.90 | | 17.78 | | 22.34 | | 34.21 | | 28.57 | | 14.29 | | 11.5 (9.8) | **< 0.01** |
| **Viral Encephalitis** | 1.85 | - | - | | 0.65 | | - | | 0.46 | | 0.73 | | 0.56 | | 0.30 | | 2.06 | | 0.70 | | 1.91 | | 2.76 | | 2.47 | | 4.17 | | 9.27 | | 9.95 | | 10.92 | | 25.42 | | 7.69 | | 3.9 (6.1) | **< 0.01** |

**Abbreviations**: CFR = Case Fatality Rate; SD = Standard Deviation

**Figure Legends**

# **eFigure 1: Flowchart of hospitalizations for meningitis and encephalitis (2012 - 2021)**

Flowchart illustrating the systematic identification and classification of the study population derived from the Swiss Federal Statistical Office. All hospitalizations with ICD-10-GM codes for meningitis and encephalitis were initially included. Cases with a length of stay (LOS) exceeding 100 days were excluded to limit the influence of extreme outliers. The final study cohort comprised *n = 23,426* hospitalizations, which were categorized into meningitis (*n = 10,160*) and encephalitis (*n = 13,266*). Subsequently, cases were stratified by infection category (bacterial, viral, or unspecified) and further classified according to pathogen-specific etiologies. This structured approach enabled the analysis of temporal trends, age-specific patterns, and clinical outcomes. Abbreviations: *B. burgdorferi* = *Borrelia burgdorferi*; *M. tuberculosis* = *Mycobacterium tuberculosis; N. meningitidis* = *Neisseria meningitidis*; *S. pneumoniae* = *Streptococcus pneumoniae*; *spp.* = species pluralis.

# eFigure 2: Pathogen categories over time and across age groups

Overview of incidence rates (IRs, per 100,000 inhabitants) for meningitis and encephalitis, categorized by pathogen type (bacterial, viral, and unspecified) and visualized using LOWESS-smoothed curves. In panel A temporal trends are shown: Annual incidence rates for meningitis and encephalitis from 2012 to 2021. In panel B age-specific patterns are shown: Incidence rates for meningitis and encephalitis across different age groups. *Abbreviations: IR = incidence rate (per 100,000 inhabitants); LOWESS = locally weighted scatterplot smoothing.*

**Alt-Text:**

**eFigure 1**. Flowchart illustrating the selection and pathogen classification of 23,426 meningitis and encephalitis hospitalizations in Switzerland (2012–2021) from a total of over 14 million records. Cases are divided into meningitis (43.4%) and encephalitis (56.6%). Within meningitis, viral pathogens predominate (55.7%), led by Enterovirus (32.1%), while bacterial pathogens (28.0%) are primarily driven by Streptococcus pneumoniae. In encephalitis, viral pathogens (48.0%) are the most common identified group, significantly dominated by Tick-borne encephalitis virus (43.9%) and herpes simplex virus (15.8%). Bacterial encephalitis is rare (9.2%). Notably, a high proportion of cases remain without pathogen specification, in encephalitis (42.8%) and meningitis (16.4%)

**eFigure 2**. LOWESS-smoothed curves illustrating incidence rates for bacterial, viral, and unspecified meningitis and encephalitis. Panel A details temporal trends from 2012 to 2021, showing a steady rise in overall incidence peaking in 2019, followed by a sharp decline in 2020–2021 primarily driven by a drop in viral meningitis and encephalitis cases. Panel B highlights age-specific patterns: viral infections show a prominent peak in young children, whereas bacterial infections exhibit characteristic bimodal distribution with higher rates in infants and the elderly.
