# Supplementary figures and images for: Nationwide Trends in Hospitalizations and Clinical Outcomes for Meningitis and Encephalitis: A 10-year Swiss Population-based Study
Source: Open Forum Infect Dis. 2026 Jun 5;13(6):ofag350. doi: 10.1093/ofid/ofag350 (PMC13263528; doi:10.1093/ofid/ofag350)

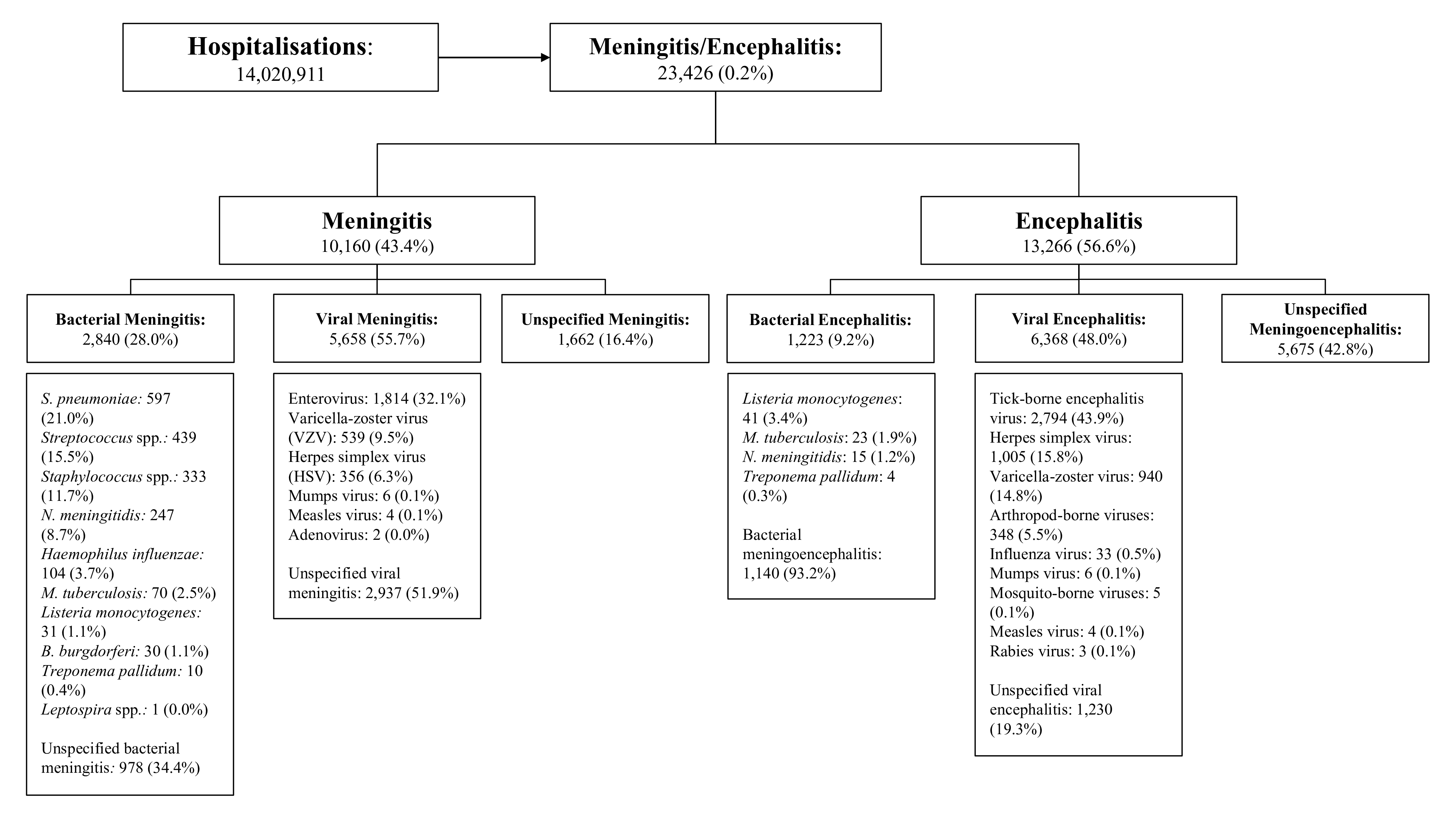

Supplement: ofag350_Supplementary_Data [file ofag350_supplementary_data.zip › eFigure 1 Flowchart.tiff]

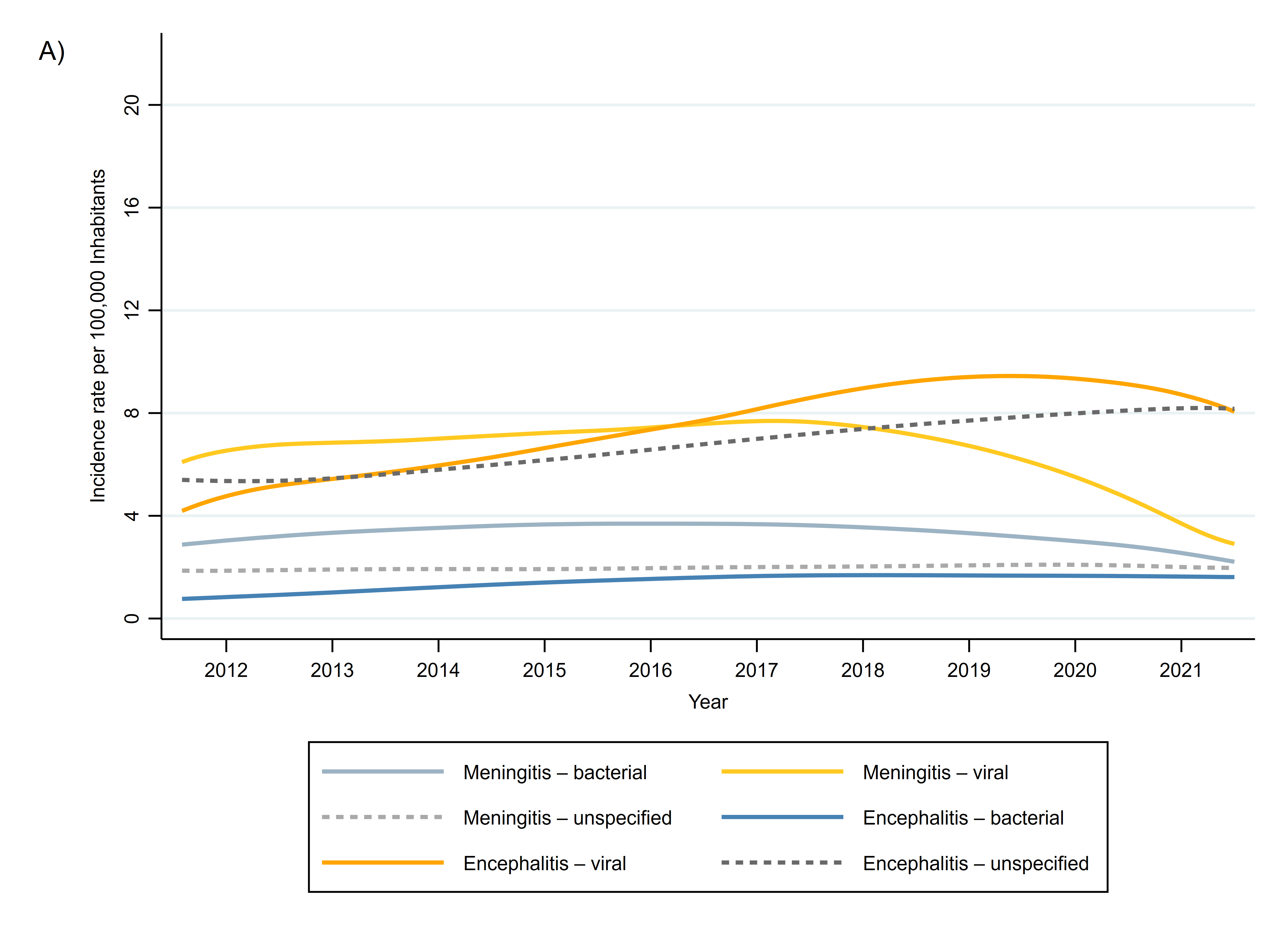

Supplement: ofag350_Supplementary_Data [file ofag350_supplementary_data.zip › eFigure2acombined_dpi400.tif]

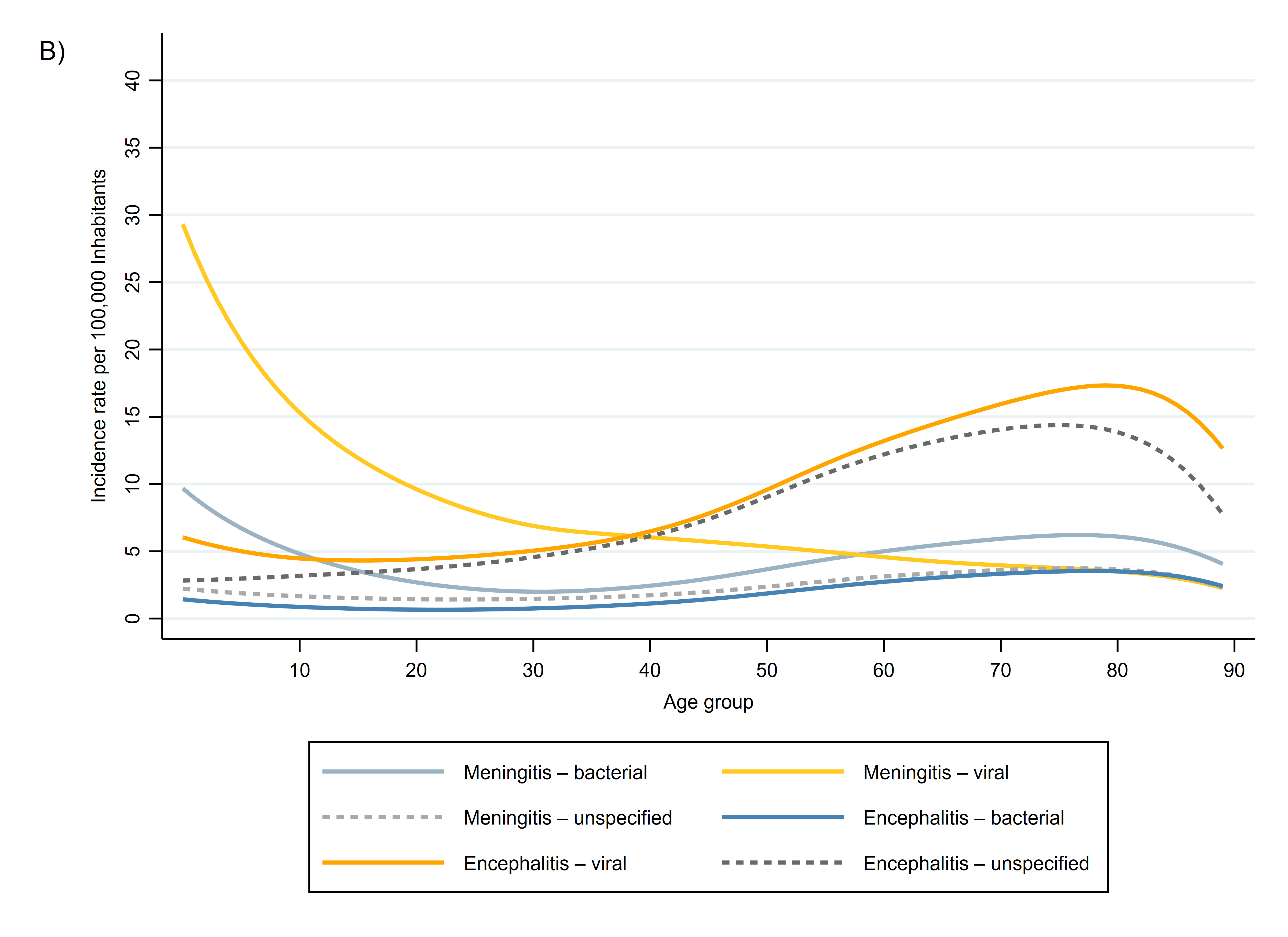

Supplement: ofag350_Supplementary_Data [file ofag350_supplementary_data.zip › eFigure2bcombined_dpi400.tif]
